# Current perspectives and trends on the role of mitochondria in renal ischemia-reperfusion injury from 2005 to 2024: a bibliometric analysis and literature review

**DOI:** 10.3389/fphys.2025.1705821

**Published:** 2025-12-10

**Authors:** Rui Yan, Yalong Zhang, Zewen Li, Kunpeng Li, Jiangwei Man, Li Yang

**Affiliations:** 1 Department of Urology, The Second Hospital of Lanzhou University, Lanzhou, China; 2 Gansu Province Clinical Research Center for Urinary System Disease, Lanzhou, China; 3 The Second Clinical Medical College, Lanzhou University, Lanzhou, China

**Keywords:** renal ischemia-reperfusion injury, mitochondrial quality control, mitochondrialmetabolic reprogramming, bibliometrics, targeted therapy

## Abstract

**Background:**

Renal ischemia-reperfusion injury (RIRI) represents a leading cause of acute kidney injury (AKI). Mitochondria, serving as the central organelles for cellular energy metabolism and signal transduction, play a pivotal role in the pathogenesis of RIRI.

**Methods:**

Utilizing the three major academic databases—Web of Science Core Collection (WoSCC), PubMed, and Scopus, this study conducted a comprehensive bibliometric analysis and visualization to explore research trends and key thematic areas related to mitochondrial involvement in renal ischemia-reperfusion injury from 2005 to 2024.

**Results:**

Bibliometric analysis reveals a sustained increase in research output concerning mitochondrial roles in RIRI over the past two decades. China and the United States have emerged as the most active contributors in this field. The U.S. Department of Veterans Affairs leads in terms of total publications, while Dong Zheng from the Second Xiangya Hospital of Central South University has contributed the highest number of publications by an individual author. Kidney International and the Journal of the American Society of Nephrology are the most frequently cited journals. This study systematically identified key research themes, including the mechanisms of mitochondrial dysfunction in RIRI, mitochondrial quality control mechanisms, and potential therapeutic strategies targeting mitochondria.

**Conclusion:**

Through bibliometric analysis, this study elucidates the knowledge structure and developmental trends in mitochondrial research related to RIRI. Over the past 20 years, mitochondrial dysfunction, mitochondrial quality control, and mitochondria-targeted therapeutic approaches have consistently constituted major research hotspots in this domain.

## 1 Introduction

RIRI is a prevalent and severe pathophysiological phenomenon in clinical practice. It commonly occurs in various clinical scenarios, including kidney transplantation, partial nephrectomy, recanalization of renal artery stenosis, and cardiovascular surgery (Zorrilla-Vaca et al., 2018). The underlying pathophysiological mechanism involves the failure of complete tissue repair following the restoration of blood flow to the kidneys after a transient ischemic period. Instead, further cellular damage ensues due to the synergistic effects of multiple factors, such as excessive reactive oxygen species (ROS) production, inflammatory mediator release, and calcium overload (Wu et al., 2018). RIRI not only contributes to the development of AKI, but also plays a pivotal role in the progression of chronic kidney disease (CKD) (Leung et al., 2013). In the context of kidney transplantation, RIRI is strongly associated with delayed graft function and reduced long-term graft survival rates (Zhao et al., 2018). Consequently, understanding the mechanisms underlying the occurrence and progression of RIRI, as well as developing effective preventive and therapeutic strategies, has become a central and challenging focus in nephrology research. From a pathogenic perspective, RIRI is not attributable to a single causative factor but rather results from the interplay of multiple pathological processes, including energy metabolism dysfunction, oxidative stress, inflammation, apoptosis, necrosis, and immune activation (Li C. et al., 2024; Decuypere et al., 2015; Chen and Yang, 2025). This complex and dynamic pathophysiological process renders RIRI a key area of basic research and a critical clinical challenge that demands urgent resolution.

Mitochondria are key organelles for energy metabolism and signal transduction within cells, playing a core role in the occurrence and development of RIRI (Pavlović et al., 2025). IRI leads to mitochondrial structural damage and functional disorders, including ATP synthesis failure, excessive production of ROS, abnormal opening of mitochondrial permeability transition pore (mPTP), mitochondrial DNA damage, etc. These events eventually trigger apoptosis, necrosis and even broader inflammatory responses in renal tubular epithelial cells (TECs) (Ma et al., 2014; Zhao M. et al., 2021). In recent years, research on mitochondria in RIRI has become a hot topic. By regulating mitochondrial function, it is expected to develop new RIRI treatment strategies.

Bibliometrics is a research tool based on quantitative analysis and statistical methods (Ma et al., 2021). By systematically sorting out the publication volume, citation status, author distribution, institutional cooperation, and evolution of research topics of academic literature, it aims to reveal the development trends and academic landscape of specific research fields (Wei et al., 2022). Bibliometrics utilizes large-scale literature data in databases and combines visualization tools to visually present the dynamic evolution of research networks, interdisciplinary studies, and keyword clustering, providing researchers with a panoramic understanding (Pan et al., 2018).

Although research results on the role of mitochondria in RIRI are increasingly abundant, most of the current related reviews focus on specific molecular pathways, cell death mechanisms or potential intervention strategies, lacking a systematic quantitative analysis of the overall research landscape and development trends. This study aims to conduct a comprehensive search and visual analysis of mitochondrial-related research literature in RIRI from 2005 to 2024 through bibliometric analysis methods. Through a systematic interpretation of the changing trends in the number of published articles, the distribution of countries and institutions, core author groups, journal influence, and keyword clustering, the development trends in this field are depicted, the research hotspots in this field are analyzed, and the emerging research trends and future development directions are explored. Through the visual analysis of relevant literature, valuable references are provided for future research, and new ideas are offered for the clinical treatment of RIRI.

## 2 Materials and methods

### 2.1 Data collection

**FIGURE 1:**
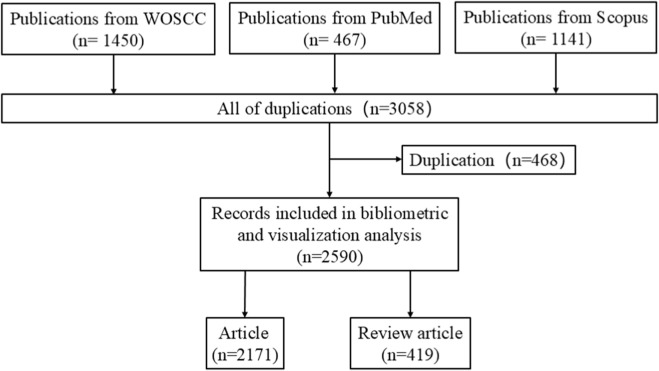
The flow chart of literature retrieval of the study.

This study employed bibliometric methods to systematically analyze the research trends and hotspots related to mitochondria in RIRI. The data sources include three major mainstream databases: the Web of Science (WOS) core Collection, PubMed and Scopus. The specific search strategies are presented in Supplementary Material S1. The search was conducted from January 2005 to December 2024 to minimize any potential discrepancies caused by daily updates. To enhance the accuracy of the data, the literature type was limited to “Article” or “Review”, and the language was restricted to English. All metadata information (including title, author, institution, keywords, abstract, publication source, citation frequency, etc.) of the obtained literature came from the three major databases respectively, and exported it in a common format (such as. txt or. ris) for subsequent data merging and analysis. To remove duplicate records, the “Deduplication” function of CiteSpace 6.4. R1 software was used for initial cleaning, and two researchers manually proofread the duplicate literature respectively to ensure the uniqueness and accuracy of the final included data. A total of 2590 literatures were ultimately included, including 1,442 from WOS, 71 from PubMed, and 1,077 from Scopus. Among them, 2,171 are Articles and 419 are reviews. The literature retrieval and screening process is shown in Figure 1.

### 2.2 Data analysis

Bibliometric analysis was conducted using CiteSpace version 6.4. R1. The time slice set for this study is 1 year, covering the period from 2005 to 2024. The node type should be selected based on the analysis purpose, including “Country”, “Institution”, “Author”, “Keyword”, “Reference”, and “Cited Journal”, etc. In the visualization graph, nodes represent specific analysis units (such as authors, keywords, etc.), and the size of nodes is positively correlated with their frequency. The lines connected indicate co-occurrence, co-citation or cooperative relationships among different nodes, with thicker lines indicating stronger associations. To identify research hotspots and cutting-edge trends, keyword co-occurrence analysis, burst keywords detection, and analysis of highly cited and frequently co-cited literature were carried out respectively, and cluster labels were generated based on clustering algorithms to explore the evolution path of disciplinary topics. The Centrality of a node is used to measure its bridging role in a network. Nodes with a value greater than 0.1 are considered to have a strong influence in the network.

## 3 Result

### 3.1 Analysis of annual publication distribution

**FIGURE 2:**
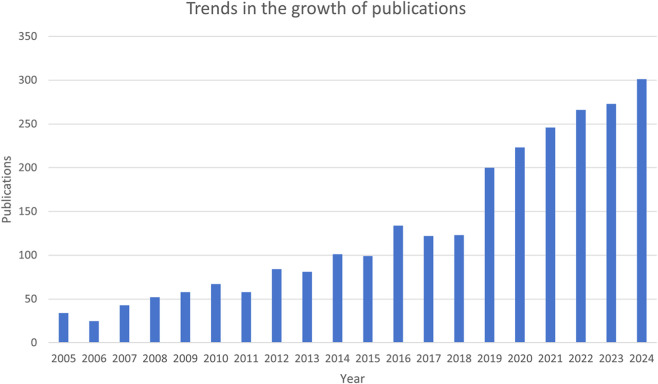
Trends in the growth of publications.

The number of publications is an indicator of the development trajectory and level of interest in the research field. According to our search strategy, the annual number of publications of mitochondria-related studies in RIRI has generally continued to rise over the past 20 years (Figure 2). Initially, there were relatively few publications in the early stage of analysis, and until 2015, the annual number of published articles was still less than 100. The number of publications has been increasing year by year since 2016. This trend continued for the next decade. Since 2019, the annual number of published articles has significantly increased, jumping from 123 in 2018 to 200 in 2019, and has continued to rise from 2020 to 2024, reaching a peak of 301 in 2024.

### 3.2 Bibliometric analysis of countries/regions and institutions

**FIGURE 3:**
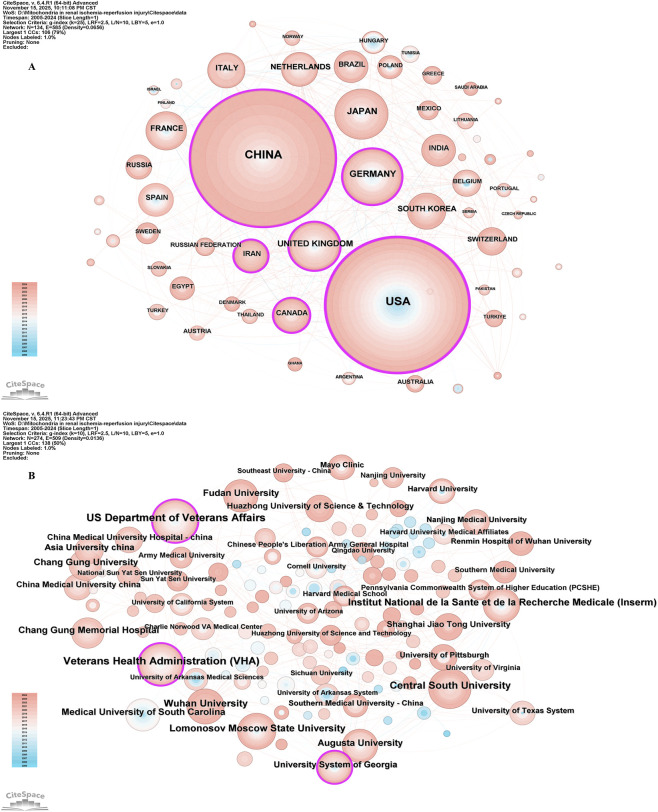
The visualization of countries regions **(A)** and institutions **(B)**. The size of nodes and edges is weighted by the number of published articles.

In the field of mitochondrial-related research in RIRI, the top ten countries in terms of the number of published papers are China, the United States, Germany, Japan, the United Kingdom, France, Italy, the Netherlands, South Korea and Brazil (Table 1). Among them, China ranked first with 744 papers, while the United States came in second with 701. The total number of papers published was significantly higher than that of other countries, which together accounted for more than 50% of the total number of papers published, demonstrating its dominant position in this field. From the perspective of the Betweenness Centrality index, the United States (0.31) is much higher than other countries, indicating that it is in an important bridge position in the international scientific research cooperation network and connects the cooperative relationships of multiple countries. Although China has the highest number of published documents, its intermediary centrality (0.18) is lower than the United States, suggesting that there is still room for improvement in the breadth of international cooperation and the core degree of its network. The United Kingdom (0.15) and Germany (0.10) also play a strong mediating role in the cooperative network, possibly related to their active multi-center clinical research and interdisciplinary cooperation (Figure 3A).

**TABLE 1:** The top 10 countries related to the research field.

Rank	Country	Frequency	Centrality
1	CHINA	744	0.18
2	United States	701	0.31
3	GERMANY	137	0.1
4	JAPAN	129	0.02
5	UNITED KINGDOM	119	0.15
6	FRANCE	85	0.05
7	ITALY	76	0.0
8	NETHERLANDS	70	0.02
9	SOUTH KOREA	67	0.03
10	BRAZIL	62	0.02

In the field of mitochondrial related research in RIRI, the top ten institutions in terms of publication volume included the US Department of Veterans Affairs, Veterans Health Administration (VHA), Central South University, Lomonosov Moscow State University, Institut National de la Sante et de la Recherche Medicale (Inserm), Augusta University, Wuhan University, Medical University of South Carolina, Fudan University and Chang Gung University (Table 2). In terms of the number of publications, institutions in the United States occupied the top position, especially the US Department of Veterans Affairs (n = 63) and the VHA (n = 61), two major systemic medical and scientific research institutions. Their research focused on the mechanisms and intervention strategies of renal allograft protection, elderly renal impairment and mitochondrial dysfunction. Central South University in China (n = 44) ranked third in the number of publications and had the highest mediation centrality (0.14), indicating that it not only has a high scientific research output, but also has a strong bridge role in the international cooperation network (Figure 3B).

**TABLE 2:** The top 10 institutions related to the research field.

Rank	Organization	Counts	Country	Centrality
1	US department of veterans affairs	63	United States	0.12
2	Veterans health administration (VHA)	61	United States	0.12
3	Central south university	44	China	0.14
4	Lomonosov Moscow State university	41	Russia	0.04
5	Wuhan university	38	China	0.03
6	Augusta university	38	United States	0.02
7	Institut national de la sante et de la recherche medicale (inserm)	38	France	0.1
8	Medical university of South Carolina	37	United States	0.08
9	Fudan university	32	China	0.03
10	Chang gung university	31	China	0.01

### 3.3 Bibliometric analysis of authors and co-cited authors

**FIGURE 4:**
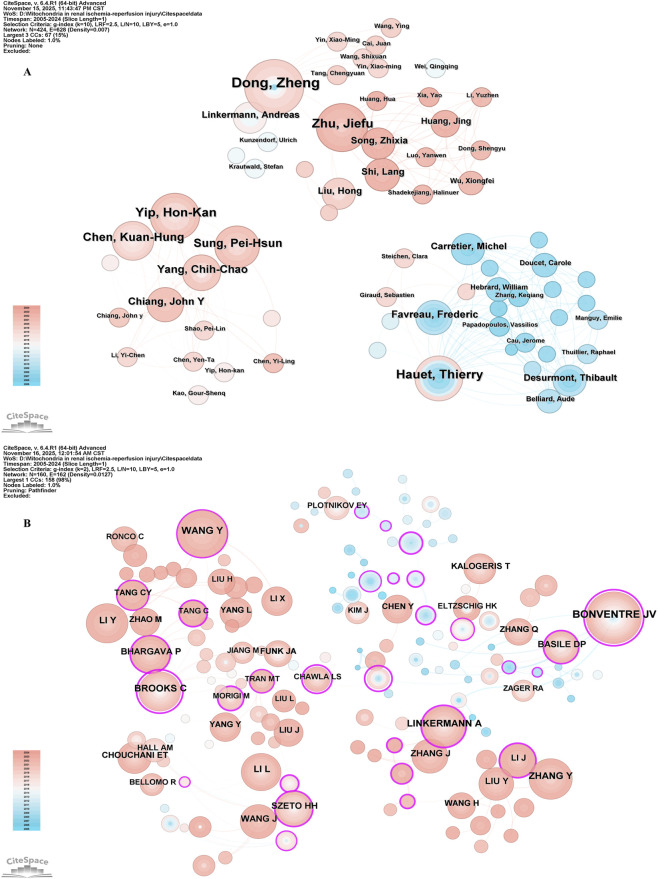
The visualization of authors **(A)** and co-cited authors **(B)**. The size of nodes and edges is weighted by the number of published articles.

A total of 11041 authors participated in the study of mitochondrial in RIRI. Table 3 lists the authors who issued the top 10 documents. The author with the highest number of published articles is Dong Zheng from the Second Xiangya Hospital of Central South University (25 articles), followed by Yip Hon-Kan from Chang Geng Memorial Hospital of Kaohsiung Medical Center in Taiwan (n = 18) and Jiefu Zhu from Central South University (n = 16). Figure 4A presents the collaborative network among different authors.

**TABLE 3:** The top 10 author related to the research field.

Rank	Author	Counts
1	Dong, zheng	25
2	Eirin, alfonso	19
3	Yip, hon-kan	18
4	Hauet, thierry	17
5	Zhu, jiefu	16
6	Sung, pei -hsun	14
7	Chen, kuan-hung	13
8	Chiang, john Y	13
9	Schnellmann, rick G	11
10	Linkermann, andreas	11

Among all the co-cited authors, there are 34 who have been cited more than 100 times. Table 4 lists the top 10 authors with the highest total citations. The author with the most citations was Bonventre JV (n = 327), followed by Wang Y (n = 249) and Brooks C (n = 198). Figure 4B presents the collaborative network among co-cited authors.

**TABLE 4:** The top 10 cited authors related to the research field.

Rank	Co-cited-author	Counts
1	BONVENTRE JV	327
2	WANG Y	249
3	BROOKS C	198
4	ZHANG Y	191
5	LI Y	176
6	LINKERMANN a	171
7	SZETO HH	162
8	LI L	156
9	BASILE DP	153
10	WANG J	147

### 3.4 Bibliometric analysis of co-cited journals and co-cited references

**FIGURE 5:**
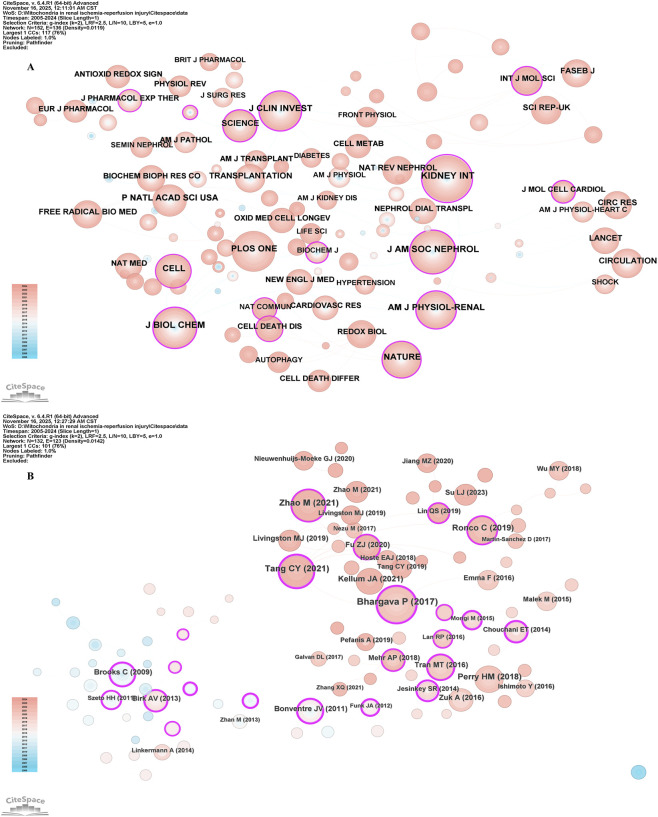
The visualization of the co-cited network of journals **(A)**; co-cited network of references **(B)**.

Table 5 lists the top 10 journals with the most co-citations. Among them, the top 5 journals are Kidney International (n = 1268) and Journal of the American Society of Nephrology (n = 1129). Journal of Biological Chemistry (n = 1000), Journal of Clinical Investigation (n = 958), PLOS ONE(n = 944) (Figure 5A).

**TABLE 5:** The top 10 co-cited journals related to the research field.

Rank	Co-cited journals	Counts
1	Kidney international	1268
2	Journal of the american society of nephrology	1129
3	Journal of biological chemistry	1000
4	Journal of clinical investigation	958
5	PLOS ONE	944
6	Nature	868
7	American journal of physiology – Renal physiology	856
8	Proceedings of the national academy of sciences of the United States of America	691
9	Cell	682
10	Science	652

Table 6 lists the top 10 most frequently cited papers. Among them, the most cited paper was Pallavi Bhargava et al. ’s review paper published in Nature Reviews Nephrology in 2017 (Bhargava and Schnellmann, 2017), followed by Meng Zhao et al.’ s original research paper published in Theranostics in 2021 (Zhao M. et al., 2021). Among the top 10 most frequently cited types of papers, 5 are original researches and 5 are review articles (Figure 5B).

**TABLE 6:** The top 10 co-cited articles related to the research field.

Rank	First author	Title	Source	Year	Counts	Centrality	Paper type
1	Pallavi bhargava	Mitochondrial energetics in the kidney	Nature reviews nephrology	2017	57	0.54	Review
2	Meng zhao	Mitochondrial ROS promote mitochondrial dysfunction and inflammation in ischemic acute kidney injury by disrupting TFAM-mediated mtDNA maintenance	Theranostics	2021	47	0.11	Article
3	Chengyuan tang	Mitochondrial quality control in kidney injury and repair	Nature reviews nephrology	2021	46	0.58	Review
4	Claudio ronco	Acute kidney injury	Lancet	2019	37	0.13	Review
5	Heather M perry	Dynamin-related protein 1 deficiency promotes recovery from AKI	Journal of the american society of nephrology	2018	34	0.07	Artical
6	John A kellum	Acute kidney injury	Nature reviews disease primers	2021	33	0	Review
7	Mei T tran	PGC1α drives NAD biosynthesis linking oxidative metabolism to renal protection	Nature	2016	27	0.28	Article
8	ZongJie fu	HIF-1α-BNIP3-mediated mitophagy in tubular cells protects against renal ischemia/reperfusion injury	Redox biology	2020	27	0.18	Article
9	Anna zuk	Acute kidney injury	Annual review of medicine	2016	25	0	Review
10	Craig brooks	Regulation of mitochondrial dynamics in acute kidney injury in cell culture and rodent models	Journal of clinical investigation	2009	23	0.57	Article

### 3.5 Bibliometric analysis of keywords

**FIGURE 6:**
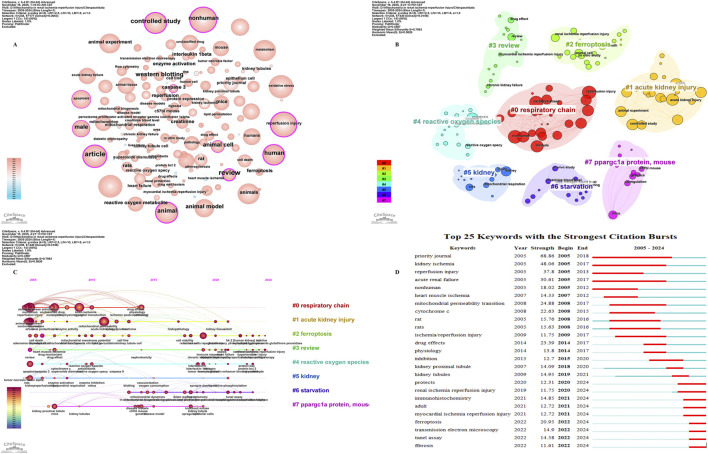
The keyword co-occurrence network **(A)**; the keywords cluster analysis **(B)**; The timeline viewer of keywords **(C)**. Top 25 keywords with the strongest citation bursts **(D)**. The size of nodes and edges is weighted by the number of published articles. The colors of the nodes represent different clusters **(A)**. All of the keywords could be classified into 8 categories, which were respiratory chain, acute kidney injury, ferroptosis, review, reactive oxygen species, kidney, starvation, ppargc1a protein, mouse **(B)**. The years between “beginning” and “end” represent periods when keywords were more influential. Years in light green indicate that the keyword has not yet appeared, years in dark green indicate that the keyword has less influence, and years in red indicate that the keyword has more influence **(D)**.

Explore the frequency and co-occurrence of keywords through Citespace. Table 7 shows the top 10 keywords. The most common keywords are reperfusion injury (n = 883), followed by nonhuman (n = 872), oxidative stress (n = 830), article (n = 706), and animals (n = 703). The keyword co-occurrence network is shown in Figure 6A. Subsequently, we conducted a cluster analysis of the keywords. All the keywords could be divided into 18 clusters, including respiratory chain, acute kidney injury, ferroptosis, review, reactive oxygen species, kidney, starvation, ppargc1a protein, mouse (Figure 6B). In addition, we created a keyword timeline graph (Figure 6C), which shows the development of keywords in each cluster. Figure 6D shows the 25 keywords with the highest outbreak rates. Explosive keywords are defined as those keywords that occur significantly within a specific time frame. This analysis not only highlights the evolution of research hotspots over time, but also reflects the latest research trends and may predict future directions. The keywords with the highest outbreak intensity are priority journal (68.86), kidney ischemia (48.06), reperfusion injury (37.8), acute renal failure (30.61) and nonhuman (18.02).

**TABLE 7:** The top 10 keywords related to the research field.

Rank	Keywords	Counts	Centrality
1	Reperfusion injury	883	0.33
2	Nonhuman	872	0.29
3	Oxidative stress	830	0
4	Article	706	0.25
5	Animals	703	0.06
6	Acute kidney injury	676	0
7	Controlled study	624	0.24
8	Metabolism	598	0.08
9	Apoptosis	591	0.13
10	Human	577	0.38

## 4 Discussion

RIRI is an important pathological basis for the progression of AKI and CKD. Renal TECs are rich in mitochondria and highly dependent on energy-dependent processes such as ion transport, reabsorption and acid-base regulation. Therefore, they are particularly sensitive to energy metabolism disorders during ischemia-reperfusion, highlighting the central role of mitochondrial dysfunction in RIRI (Huang et al., 2024). In this study, we conducted a bibliometric analysis of mitochondrial-related research in RIRI.

### 4.1 General information

The number of publications in the field of mitochondria in RIRI has continued to increase over the past 20 years. From 2018 to 2019, the annual number of published articles jumped significantly and continued to grow in the next 5 years. The rapid growth at this stage may be attributed to the following reasons. Firstly, the development of high-throughput technologies capable of detecting differences in various molecular components in organisms (including genomics, transcriptomics, proteomics, metabolomics and epigenomics), namely high-throughput omics technologies, has made significant contributions to clarifying the structure and function of mitochondria, as well as the disease impacts of mitochondrial dysfunction (Rahman and Rahman, 2018). Secondly, the application of a series of novel imaging techniques capable of ultrastructural scanning provides a methodological basis for mitochondrial research (Okumura et al., 2018; Ohta et al., 2021). It simultaneously reflects the urgency of clinical needs such as the protection of transplanted kidneys, the prevention and treatment of AKI, as well as the deepening understanding of the role of mitochondria in RIRI by the international academic community.

We have observed that China and the United States occupy leading positions in the field of mitochondrial research in RIRI, reflecting the advantages of their continuous financial investment, complete scientific research systems and international cooperation networks in the field of biomedical research. The US Department of Veterans Affairs has the most research achievements in this field, which indicates that the institution has remarkable scientific research ability and focus in RIRI research. These results reveal the global geographical distribution of mitochondrial research and the concentrated distribution of research productivity in RIRI, and also highlight the importance of international cooperation in promoting progress in this field.

Overall, the high-yield author groups are distributed in the United States, China and France. Their research directions cover not only the exploration of the basic mechanisms of mitochondrial structure and function, but also clinical translational research on stem cells, drugs and organ protection, presenting the characteristics of equal emphasis on mechanism research and clinical application, as well as significant cross-border cooperation. This group not only promoted the rapid development of mitochondrial research in RIRI, but also played a key role in the international cooperation network.

The most frequently cited journals are Kidney International and Journal of the American Society of Nephrology, both of which are top professional journals in the field of nephrology, indicating that the research topic is highly focused on the core areas of nephrology. Meanwhile, top comprehensive or basic medical journals such as Journal of Biological Chemistry, Journal of Clinical Investigation, Cell, Nature and Science have been cited extensively. This indicates that research in this field not only focuses on clinical application but is also driven by fundamental molecular mechanism and cell biology studies.

### 4.2 Analysis of research hotspots based on keywords and co-cited literature

Bibliometric analysis clearly Outlines the dynamic evolution trajectory of mitochondrial research focus in the field of renal ischemia-reperfusion injury over the past two decades. Analysis shows that the research paradigm in this field exhibits distinct phased characteristics. In the early stage (approximately 2005–2012), the research focus was mainly concentrated on explaining the fundamental damage mechanism of mitochondria in RIRI. Scholars are dedicated to revealing core events such as the explosive accumulation of oxidative stress, the release of cytochrome c, the abnormal opening of mitochondrial permeability transition pores, and the triggered caspase-dependent apoptotic pathway. These pioneering works have laid the foundation for our understanding of the core pathophysiological role of mitochondria in RIRI and established its position as a key hub for cell fate decision-making. It is worth noting that since around 2015, the research perspective in this field has undergone a significant strategic shift. The research focus has systematically expanded from the description of isolated damage phenomena to the exploration of dynamic regulatory networks and their transformation potential. This transformation is prominently reflected in the following several cutting-edge directions: First, the mitochondrial quality control system, including dynamic-associated protein 1 (DRP1)-mediated mitochondrial over-fission, mitochondrial fusion imbalance regulated by Mitofusin-1/2 (MFN1/2) and Optic atrophy 1 (OPA1), and mitophagy driven by the PTEN-induced putative kinase 1 (PINK1)/Parkin signaling axis; Secondly, energy metabolism reprogramming. Research has begun to focus on how cells are forced to shift from efficient fatty acid oxidation to inefficient glycolysis under ischemic conditions and its profound impact on cell survival. Thirdly, new therapeutic strategies targeting mitochondria, such as mitochondrial-targeted antioxidants (Mito-Tempo, MitoQ) and drug interventions aimed at precisely regulating key nodes of mitochondrial quality control, have become new research hotspots.

Through in-depth analysis of keyword emergence, co-citation clustering of literature, and high-impact papers, we have consistently observed a clear migration path of research hotspots: that is, gradually moving from the primary stage of “describing structure and damage” to the advanced stage of “intervention regulation and precise treatment”. This transformation not only reflects the deepening understanding of the RIRI mechanism by the academic community, but also demonstrates the urgent desire to transform basic research findings into clinical applications.

Based on the distinct trends revealed by the above bibliometric analysis, the following text will first systematically discuss “mitochondrial dysfunction in RIRI”, and then deeply analyze “the key links that drive the occurrence and progression of diseases through mitochondrial quality control and mitochondrial metabolic reprogramming”. Based on this, we will focus on discussing the latest progress of “potential therapeutic strategies targeting mitochondria” and look forward to the cutting-edge research directions with clinical translation potential in the future.

#### 4.2.1 Mitochondrial dysfunction in RIRI

Mitochondria play a core role in cellular energy metabolism and are also important organelles for maintaining the balance of ROS and Ca^2+^. Therefore, mitochondrial dysfunction is a core link in RIRI.

**FIGURE 7:**
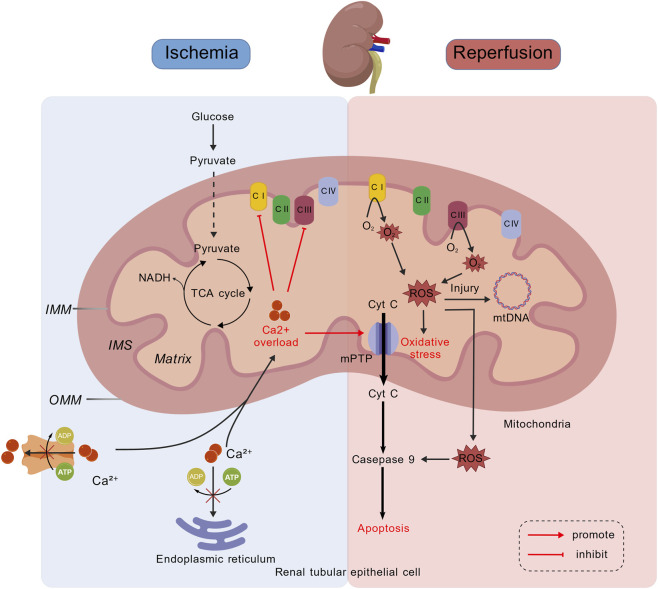
Mitochondrial dysfunction in renal ischemia-reperfusion injury. RIRI begins with energy metabolism disorders and ionic homeostasis imbalance during the ischemic period. During ischemia, aerobic respiration in mitochondria is inhibited and ATP synthesis in cells is impaired. The reduction of ATP leads to intracellular Ca overload, which further inhibits mitochondrial oxidative phosphorylation. During the reperfusion period, ROS are generated in an explosive manner, and the intracellular calcium overload instrument induces the opening of mPTP, triggering mitochondrial-mediated apoptosis. These processes interweave and amplify each other, forming a complex damage network.

After the blood flow is interrupted, the renal TECs and interstitial cells fall into a state of hypoxia, the activity of various complexes in the electron transport chain decreases, mitochondrial aerobic respiration is inhibited, and cellular metabolism shifts from efficient oxidative phosphorylation to anaerobic glycolysis (Kosieradzki and Rowiński, 2008). This will lead to ATP production disorders and prevent the normal life activities of cells from being maintained. During ischemia, the reduction of ATP synthesis leads to the obstruction of cytoplasmic Ca^2+^ excretion and endoplasmic reticulum reuptake, resulting in cytoplasmic Ca^2+^ overload. Mitochondria are forced to take in excessive Ca^2+^, which leads to an overload of Ca^2+^ in the mitochondrial matrix (Halestrap, 2006). Excessive Ca^2+^ will directly inhibit the functions of electron transfer chain (ETC.) complexes I and III, further limiting mitochondrial oxidative phosphorylation (Prasad Panda and Kesharwani, 2023). In addition, ischemia and Ca^2+^ overload make the mPTP sensitive to openings. mPTP is a non-specific highly conductive channel located in the inner mitochondrial membrane. The continuous opening of mPTP will lead to the loss of mitochondrial membrane potential and further block the production of ATP (Griffiths and Halestrap, 1995). Meanwhile, mitochondrial contents, including Cytochrome c (Cyt c) and apoptosis-inducing factors, are released, activating downstream endogenous apoptotic programs and thereby inducing cell death (Javadov et al., 2011). Oxidative stress imbalance is the most prominent feature of RIRI, especially during the reperfusion stage. During the ischemic period, the function of the ETC complex is inhibited and nicotinamide adenine dinucleotide (NADH) accumulates (Kosieradzki and Rowiński, 2008; Sammut et al., 2000). After reperfusion, a sudden large supply of oxygen occurred, and the ETC., function failed to recover in time, resulting in a large number of electrons leaking out. These electrons combined with O_2_ to form superoxide anions (O_2_•^-^), triggering an “explosive” generation of ROS (Granger and Kvietys, 2015). Meanwhile, IRI consumes antioxidant substances within cells, leading to an imbalance in the antioxidant defense system. Excessive ROS not only directly attacks mtDNA, lipids and proteins, destroying the structure and function of mitochondria, but also acts as signal molecules to activate multiple inflammatory and apoptotic pathways, forming a vicious cycle and exacerbating cell damage (Figure 7) (Rovcanin et al., 2016; Granata et al., 2022).

#### 4.2.2 Mitochondrial quality control in RIRI

In the RIRI process, mitochondrial dysfunction is considered a core component. Mitochondrial morphology and functional homeostasis depend on complex and dynamic mitochondrial quality control mechanisms. The process of mitochondrial quality control primarily involves three mechanisms: mitochondrial biogenesis, mitochondrial dynamics (fusion and fission), and mitophagy, which protect mitochondria from damage and prevent the accumulation of defective mitochondria (Liu B. H. et al., 2024). In RIRI, these mechanisms are interconnected and synergistically regulate renal stress responses and protection. Figure 2 shows mitochondrial quality control in renal ischemia reperfusion injury.

##### 4.2.2.1 Mitochondrial biogenesis in RIRI

Mitochondrial biogenesis is a self-renewal process initiated by cells to meet energy demands and respond to environmental stress (Popov, 2020). It depends on the coordinated regulation of the nuclear genome and the mitochondrial genome (Liu et al., 2023). Peroxisome proliferator-activated receptor gamma coactivator 1 alpha (PGC-1α) is recognized as a key regulator of this process (Halling and Pilegaard, 2020). Its activation occurs through phosphorylation by Adenosine 5‘-monophosphate-activated protein kinase (AMPK) or deacetylation by Silent Information Regulator 1(SIRT1) (Yuan et al., 2016; Jeon, 2016), subsequently driving downstream transcription factors such as nuclear respiratory factor 1/2 (NRF1/NRF2) (Zhao T. et al., 2023; Esteras et al., 2022) and estrogen-related receptor alpha (ERR-α) (Fan et al., 2025), which in turn promote the expression of mitochondrial transcription factor A (TFAM). TFAM is responsible for mtDNA replication and transcription (Kunkel et al., 2016). While some mitochondrial proteins are encoded by mtDNA and translated within mitochondria, the vast majority (approximately 60%–95%) are encoded by nuclear genes (Moulin et al., 2019). These proteins are synthesized on cytoplasmic ribosomes and subsequently imported into the mitochondrial matrix or inner membrane via transport systems (e.g., the TIM23 complex) (Sim et al., 2023).

In RIRI, mitochondrial biogenesis serves as a critical restorative mechanism for reestablishing cellular energy metabolism and function, governed by multidimensional precision regulation. Its core regulator, PGC-1α, is frequently downregulated in kidneys following IRI, involving p53-mediated transcriptional inhibition. Downregulation of PGC-1α leads to impaired mitochondrial function and ATP depletion (Wang et al., 2025). Upregulating PGC-1α promotes mitochondrial DNA replication, electron transport chain complex protein expression, and restoration of oxidative phosphorylation by activating downstream factors such as NRF1 and TFAM, thereby improving energy metabolism and survival capacity in renal tubular cells (Shirzad et al., 2023; Wang D. et al., 2020). PGC-1α activity is regulated by multiple signaling pathways. SIRT1, an energy-sensing enzyme, activates PGC-1α through acetylation modification, thereby promoting mitochondrial biogenesis (Khader et al., 2014). The circadian rhythm gene Brain and Muscle ARNT-Like Protein 1 (BMAL1) mediates mitochondrial homeostasis in RIRI by activating the SIRT1/PGC-1α axis (Ye et al., 2022). Conversely, the increased expression of the transcription factor Twist1 will inhibit the transcription of PGC-1α and further suppress the expression of FAO-related genes (PPARα, CPT1 and ACOX1), leading to mitochondrial dysfunction (Liu et al., 2022). Furthermore, mitochondrial ROS (mtROS) released during IRI have been shown to promote Lon-mediated TFAM degradation, reducing mitochondrial TFAM abundance in TECs. TFAM deficiency further inhibits mtDNA synthesis and mitochondrial biogenesis, resulting in mitochondrial DNA depletion and respiratory defects that exacerbate TEC dysfunction during IRI-AKI (Zhao M. et al., 2021).

##### 4.2.2.2 Mitochondrial dynamics in RIRI

Mitochondria are in a highly dynamic state of change within cells. Mitochondrial dynamics refers to the continuous division and fusion of mitochondria to maintain their normal shape. The characteristic of mitochondrial fission is that one mitochondrial splits into two sub-mitochondria, while mitochondrial fusion is the combination of two mitochondria to produce one mitochondrial. The relative balance between mitochondrial division and fusion is crucial for maintaining the quality and function of mitochondria and is an important foundation for ensuring the normal activities of cells.

###### 4.2.2.2.1 Mitochondrial fission in RIRI

Mitochondrial fission is a process of mitochondrial proliferation, dominated by DRP1 (Smirnova et al., 2001; Pagliuso et al., 2018). Under physiological conditions, the DRP1 protein is usually located in the cytoplasm (Kraus et al., 2017). During mitochondrial division, DRP1 is recruited to outer membrane and, under the synergistic action of receptor proteins such as MFF, MiD49 and MiD51, forms a contractile loop, promoting mitochondrial division (Ji et al., 2019) (Gandre-Babbe and van der Bliek, 2008) (Losón et al., 2013). In RIRI, the activity of DRP1 is significantly enhanced, and its recruitment to the mitochondrial membrane leads to mitochondrial rupture, loss of membrane potential and energy generation disorders (Song et al., 2024). This process is accompanied by the release of Cyt C, which further triggers apoptosis and necrosis of cells (Wang et al., 2022).

The regulation of DRP1 activity mainly relies on post-translational modifications such as phosphorylation, acetylation and ubiquitination. Studies have shown that enhanced phosphorylation of DRP1 at the Ser616 site can promote its activation and accelerate fission, while dephosphorylation at the Ser637 site also promotes the fission process (Shi et al., 2024). In RIRI, miR-199a-5p reduced the phosphorylation level at the Ser637 site of DRP1 by targeting and inhibiting AKAP1, leading to its overactivation and ultimately causing severe mitochondrial fragmentation and energy metabolism disorders (Shi et al., 2024). In animal experiments, specifically blocking the interaction between DRP1 and mitochondrial fission 1(Fis1) has been confirmed to alleviate mitochondrial lysis and cell damage in RIRI, and significantly improve renal function in ischemic models of mice and pigs (Song et al., 2024). In addition, the regulation of DRP1 activity by upstream signaling pathways is equally important. Calcium/calmodulin-dependent protein kinase II (CaM kinase II, CaMKII) has been proven to directly promote the phosphorylation of DRP1 and enhance fission. Its abnormal activation in RIRI exacerbates mitochondrial damage (Wang et al., 2022). Calcineurin regulatory protein Regulator of Calcineurin 1 (RCAN1) can activate JNK signaling and promote MFF expression, thereby enhancing the recruitment of DRP1 to the mitochondrial outer membrane, significantly aggravating mitochondrial damage and apoptosis. Meanwhile, the absence of RCAN1 can improve mitochondrial dysfunction related to RIRI (Xiao et al., 2022). These findings reveal the molecular network of DRP1 activation and suggest its key role in RIRI. Excessive fission mediated by DRP1 causes significant pathological consequences in RIRI (Huang et al., 2024), including membrane potential disintegration, electron transport chain disorders and ATP depletion, and initiates an apoptotic cascade through cytochrome c leakage (Wang et al., 2022). (Song et al., 2024). The ROS outbreak in the early stage of reperfusion further damages mtDNA and membrane structure, causing a vicious cycle of damage (Wang et al., 2024). In renal TECs, excessive fission not only drives apoptosis and necrosis, but is also associated with inflammatory responses and the progression of fibrosis (Xiao et al., 2022). Due to the fission load exceeding the autophagy clearance capacity, the accumulation of damaged mitochondria further aggravates tissue damage, suggesting that excessive fission is an important driving factor for the transformation from acute injury to chronic injury (Wang et al., 2024).

###### 4.2.2.2.2 Mitochondrial fusion in RIRI

Mitochondrial fusion achieves the structural integration of mitochondria through the sequential fusion of the outer and inner membranes and is a key process for maintaining the homeostasis of the mitochondrial network (Chan, 2020). Outer membrane fusion is mainly mediated by MFN1 and MFN2, both of which belong to the kinetic protein superfamily and drive the remodeling of the outer membrane lipid billet through GTP hydrolysis (Kijima et al., 2005; Ishihara et al., 2004). In addition to regulating fusion, MFN2 also mediates the interaction between mitochondria and endoplasmic reticulum (mitochondria-associated membranes, MAMs), playing a unique role in calcium signal transport and metabolic regulation (De Brito and Scorrano, 2008; Filadi et al., 2015). OPA1 maintains crest structure and promotes endometrial integration through the dynamic balance of long and short subtypes, and its function depends on MFN1 rather than MFN2 (Song et al., 2009; Ban et al., 2017; Tondera et al., 2009; Cipolat et al., 2004).

In RIRI, MFN2 and OPA1 are significantly downregulated in the early stage of reperfusion, leading to rapid disintegration of the mitochondrial network, decreased membrane potential and impaired ATP production (Sumida et al., 2015; Chen P. et al., 2023). As the injury progresses, mitochondrial fragmentation further intensifies, promoting the massive generation of ROS and inducing oxidative stress, thereby exacerbating cell apoptosis and necrosis (Leboucher et al., 2012). The downregulation of MFN2 is regarded as one of the causes of mitochondrial-endoplasmic reticulum communication disorders, which lead to calcium overload and endoplasmic reticulum stress, exacerbating apoptosis and inflammatory responses (Huang et al., 2024). Restoring MFN1/2 levels can partially reverse fusion disorders, enhance antioxidant capacity and reduce ROS generation (Zhan et al., 2013). The reduction of OPA1 in RIRI leads to the loss of ridge structure and energy metabolism disorders, while promoting the release of cytochrome c and the activation of the caspase pathway (Zhan et al., 2013). In addition, a decline in OPA1 has been proven to weaken mitophagy, making it difficult to clear damaged mitochondria and thereby exacerbating cell damage (Zhan et al., 2013).

The above research indicates that mitochondrial dynamics play a core role in the occurrence and development of RIRI, and its equilibrium state directly affects mitochondrial function, oxidative stress levels and cell survival. In-depth research on its molecular mechanism and exploration of corresponding intervention methods will provide new ideas and potential clinical application value for the prevention and treatment of RIRI.

##### 4.2.2.3 Mitophagy in RIRI

As a form of selective autophagy in cells, mitophagy maintains mitochondrial homeostasis by selectively eliminating damaged or excess mitochondria (Kim et al., 2007; Pickles et al., 2018). In metabolically active kidneys with high mitochondrial abundance, mitophagy is particularly crucial for limiting mitochondrial damage. During RIRI, ischemia leads to ATP depletion, and reperfusion triggers ROS bursts and a decrease in membrane potential, making mitochondria the main target of damage (Ralto and Parikh, 2016; Li et al., 2024a). Unlike non-selective autophagy, mitophagy requires specific recognition signals and molecular pathways. In mammals, the damage-sensing pathway mediated by PINK1 and E3 ubiquitin ligase Parkin is a classic ubiquitin-dependent mitophagy pathway (Ashrafi and Schwarz, 2013; Yu et al., 2011). On healthy mitochondria, the PINK1 protein is rapidly introduced and degraded (Meissner et al., 2011). However, once the mitochondrial membrane potential (ΔΨm) is lost, the introduction and degradation process of PINK1 is inhibited, resulting in its stable accumulation on the outer mitochondrial membrane (Narendra et al., 2010). Subsequently, PINK1 recruits Parkin to the mitochondrial surface, inducing extensive ubiquitination of outer membrane proteins (Lazarou et al., 2012; Okatsu et al., 2013; Koyano et al., 2014; Ordureau et al., 2014). The accumulation of ubiquitination triggers the recruitment and accumulation of autophagy receptors optineurin (OPTN) and nuclear spot protein 52 (NDP52). OPTN and NDP52 interact with microtubule-associated protein 1 light chain 3 (LC3), anchor UB-labeled mitochondria to autophagosomes, and form mitochondrial autophagosomes. Mitochondrial autophagosomes fuse with lysosomes. Eventually, under the action of various hydrolases in lysosomes, damaged mitochondria are completely degraded (Ivankovic et al., 2016).

A large number of studies have confirmed that the PINK1/Parkin pathway is activated and has a protective effect in RIRI. Depletion of PINK1 and Park2 attenuated mitophagy, thereby enhancing mitochondrial damage, ROS production, and inflammatory responses, leading to increased tubular damage and AKI after RIRI in mice (Tang et al., 2018). The protective effect of iscNImic preconditioning (IPC) also depends on the activation of PINK1 (Livingston et al., 2019). Comorbidities such as diabetes aggravate IRI by inhibiting the PINK1/Parkin, which makes the renal tubules more sensitive to ischemic injury (Yang et al., 2019). 8-oxoguanine DNA glycosidase (OGG1) is a DNA glycosylase responsible for the removal of oxidative damage 8-oxoguanine (8-oxoG) from DNA, and its expression is induced during IRI. OGG1 negatively regulates mitophagy by inhibiting PINK1/Parkin pathway, thereby mediating IRI-induced apoptosis and renal tissue injury. Knockout or pharmacological inhibition of OGG1 can alleviate RIRI by partially activating mitophagy (Zhao F. et al., 2023). In addition, certain stress states such as hyperbilirubinemia or BPA exposure can lead to excessive activation of mitophagy, which also aggravates the damage (Liao et al., 2023; Peerapanyasut et al., 2020). Some positive regulatory factors exert protective effects by targeting the PINK1/Parkin pathway. The expression of N-myc downstream regulatory gene 2 (Ndrg2) was significantly decreased after RIRI. Liu et al. found that mice with Ndrg2 gene deletion exhibited significantly reduced RIRI, and the mechanism was closely related to the reduction of oxidative stress, maintenance of mitochondrial homeostasis, and activation of PINK1/Parkin-mediated mitophagy (Liu M. et al., 2024).

In contrast, PRKN/PARK2-independent mitophagy is directly mediated by mitophagy receptors. For example, Bcl-2/adenovirus E1B 19-kDa interacting protein 3 required for red blood cell maturation in mammals and its homologous protein Nix/BCL2 Interacting Protein 3 (BNIP3), as well as hypoxia-induced FUN14 domain 1 (FUNDC1). They form a formative expression on mitochondrial outer membrane. These mitophagy receptors can directly bind to MAP1LC3B/LC3B (microtubule-associated protein 1 light chain 3 β) on autophagosome precursor phagocytes and directly recruit autophagy without ubiquitination modification (Ding et al., 2010; Liu et al., 2012; Liu et al., 2014).

In the *in vivo* and *in vitro* models of RIRI, the expression of BNIP3 increased. Silencing BNIP3 in renal tubular cells reduced IRI-induced mitophagy and aggravated H/R-induced cell death (Tang et al., 2019). Depletion of hypoxia-inducible factor-1α (HIF-1α) significantly attenuated H/R-induced mitophagy and aggravated H/R-induced apoptosis and ROS production. The overexpression of BNIP3 reversed the damage caused by HIF-1α knockout (Fu et al., 2020). The expression and function of BNIP3 are tightly regulated by a complex network. Twist-related protein 2 (Twist2) is a basic helix-loop-helix transcription factor. Twist2 directly inhibited the transcriptional activity of BNIP3 by targeting the BNIP3 promoter. (Zhang L. et al., 2025). Peroxisome proliferator-activated receptor α (PPARα) is a positive transcriptional regulator of BNIP3. Upregulation of PPARα induces the transcription of BNIP3, thereby maintaining mitochondrial homeostasis, reducing kidney injury and delaying the transition from AKI to CKD in IRI and cisplatin-induced AKI models (Yao et al., 2022). In the renal TECs induced by IRI, the expression of FUNDC1 is upregulated, promoting the activation of mitophagy (Zhang et al., 2024). Furthermore, studies based on multi-center transcriptome analysis have shown that FUNDC1 is one of the key mitophagy-related genes affecting prognosis and immune cell infiltration in the immune microenvironment of RIRI, suggesting that it not only participates in mitochondrial quality control but is also closely related to immune regulation after RIRI (Chen R. Y. et al., 2023). More importantly, Huang et al. 's research demonstrated that FUNDC1 is an indispensable molecule for the renal protective effect mediated by IPC. IPC activates mitophagy by enhancing the phosphorylation of FUNDC1 at the Ser17 site, thereby inhibiting DRP1-dependent mitochondrial fission, improving mitochondrial quality, and significantly alleviating IRI. However, in mice with proximal tubule-specific knockout of FUNDC1, the above-mentioned protective effect was completely lost (Wang J. et al., 2020).

##### 4.2.2.4 The interaction between mitochondrial dynamics and mitophagy in RIRI

Mitochondrial dynamics and mitophagy are not two independent processes, but rather a closely coupled and mutually coordinated unified system that jointly determines the quality of mitochondria and the fate of cells. The imbalance of mitochondrial dynamics will directly affect the efficiency of mitophagy.

**FIGURE 8:**
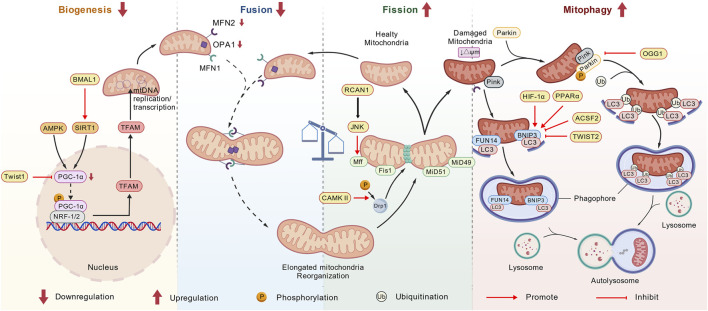
Mitochondrial quality control in renal ischemia-reperfusion injury. Mitochondrial quality control encompasses mitochondrial biogenesis, mitochondrial dynamics (mitochondrial fission and fusion), and mitophagy. In RIRI, the expression of PGC-1α, a core regulatory factor of mitochondrial biogenesis, is downregulated, leading to impaired mitochondrial function and ATP depletion. Mitochondrial fission and fusion are two interrelated dynamic processes. In RIRI, the fission protein Drp1 is overactivated, while the expression of fusion proteins MFN2 and Opa1 is downregulated. Imbalance between mitochondrial fission and fusion, excessive fission leads to apoptosis and promotes necrosis and inflammatory responses. In addition, mitophagy is activated in RIRI to clear damaged mitochondria and maintain mitochondrial homeostasis and function.

Fission separates the damaged mitochondrial fraction from the network, creating smaller units that are prone to autophagosome encapsulation on the one hand, and also helping to expose signaling molecules on the mitochondrial membrane, such as PINK1 stabilization, to initiate the mitophagy pathway on the other (Pavlović et al., 2025; Livingston et al., 2019). There is mitophagy activated by DRP1-dependent pathway in RIRI. Moderate mitophagy helps to maintain mitochondrial homeostasis, reduce ROS production and apoptosis by removing dysfunctional mitochondria (Zhao W. et al., 2021). Inhibition of DRP1 not only alleviated excessive mitochondrial fission, but also significantly inhibited mitophagy induced by IRI (Li et al., 2018). Mitochondrial fusion facilitates content sharing, compensates for minor damage, and maintains the functional integrity of the mitochondrial network. The role of MFN2 is particularly special. MFN2 is essential for mitophagy mediated by the PINK1/Parkin pathway because PINK1 stabilization and Parkin recruitment are partially dependent on MFN2 (Li et al., 2024b). However, in receptor-mediated pathways such as BNIP3, the role of MFN2 may be more complex and it may indirectly regulate calcium signaling and lipid exchange by maintaining the integrity of MAMs, thereby affecting the overall environment of mitophagy (Li et al., 2024b). OPA1 maintains the structure and energy metabolism of mitochondrial inner membrane cristae. Cleavage and loss of function of OPA1 is a hallmark of mitochondrial damage, leading to mitochondrial fragmentation and cytochrome C release, inducing apoptosis. Maintaining the functional integrity of OPA1 helps to maintain mitochondrial membrane potential and reduce ROS production, thereby potentially alleviating excessive stress on the mitophagy system (Zanfardino et al., 2025). Uncoupling protein 2 (UCP2) -dependent improvement of mitochondrial dynamics protects against AKI. UCP2 depletion aggravated hypoxia-induced mitochondrial fission, while increased UCP2 expression improved the balance between mitochondrial fusion and fission and reduced mitophagy (Qin et al., 2019). Therefore, promoting fusion (e.g., by upregulating MFN2, OPA1) helps to stabilize the mitochondrial network and increase the functional threshold of mitochondria, which may reduce the need for massive mitophagy and thus play a protective role in IRI (Figure 8) (Qin et al., 2019).

#### 4.2.3 Mitochondrial metabolic reprogramming

The metabolic processes of sugars, fatty acids and proteins within mitochondria provide 95% of the energy required for vital activities of the cell. Mitochondrial metabolic reprogramming refers to a biological process in which cells undergo fundamental changes in energy metabolism to adapt to energy demand and microenvironmental changes under stress conditions. Metabolic reprogramming involves glucose metabolism, lipid metabolism, and amino acid metabolism, regulates the metabolic pattern of living cells, and it is an evolutionarily conserved self-defense mechanism to cope with injuries such as hypoxia (Liu C. et al., 2024).

Under normal conditions, energy-consuming cells such as renal TECs mainly rely on efficient fatty acid oxidation (FAO) and oxidative phosphorylation to produce large amounts of ATP. The core of metabolic reprogramming in RIRI is the transition of energy metabolism from efficient FAO to inefficient glycolysis in TECs. This process is precisely regulated by multiple levels. TIMP metallopeptidase inhibitor 2 (TIMP2) activates the Hedgehog signaling pathway to promote DRP1-mediated mitochondrial fission and inhibit MFN2, leading to mitochondrial fragmentation and dysfunction (Pang et al., 2025). As a cellular energy factory, mitochondrial dysfunction (such as decreased membrane potential) is the initiating factor and core link of metabolic reprogramming (Pavlović et al., 2025). After renal TECs undergo IRI, the C-terminal transcriptional activation domain (CTAD) of HIF-1α is activated, which can directly upregulate the expression of hexokinase 2 (HK2) and strongly drive the glycolysis process (Zhang Y. et al., 2025). In addition, AMPK acts as an energy sensor and a central switch in determining the cell fate transition to FAO or glycolysis by regulating PGC1α-CPT1A and HIF1α-HK2 downstream pathways (Xu et al., 2023). Metabolic reprogramming is also finely regulated by protein post-translational modifications. The ubiquitin E3 ligase TRIM21 inhibits aerobic glycolysis by ubiquitin-degradation of the key glycolytic enzyme PFKP (Wen et al., 2024). The serine/threonine kinase Polo-like Kinase1 (PLK1) inhibits phosphatase activity of PTEN by phosphorylation, thereby relieving the inhibition of phosphoglycerate kinase 1 (PGK1), and finally activate the glycolysis pathway (Jiang et al., 2025). Calmodulin 2 (CNN2) is mainly expressed in fibroblasts and pericytes. CNN2 deficiency can upregulate CPT1A and other key enzymes through ESR2-PPARα axis, promote FAO and reduce renal fibrosis after RIRI (Gui et al., 2023). It is worth noting that metabolic reprogramming occurs not only in parenchymal cells, but also in immune cells. Cytidine/uridine monophosphate kinase 1 (CMPK1) driven nucleotide metabolic reprogramming in monocytes promotes mitochondrial damage, pyroptosis and inflammation, and exacerbates injury (Zhang H. et al., 2025). These changes are manifested in the metabolomics of amino acid metabolism, nucleotide metabolism and tricarboxylic acid cycle disorders, as well as lipid metabolism disorders and lactate accumulation (Yang X. et al., 2023), which together constitute a complex regulatory network of metabolic reprogramming in IRI.

#### 4.2.4 Targeting mitochondria to treat RIRI

##### 4.2.4.1 Targeting mitochondrial biogenesis

In therapeutic strategies targeting mitochondrial biogenesis, multiple drugs and interventions demonstrate significant potential for improving renal function and mitochondrial homeostasis after IRI by upregulating PGC-1α expression or enhancing its activity. The natural compound curcumin, with enhanced bioavailability via nanomedicine formulations, activates the NRF2/HO-1 and PGC-1α pathways to mitigate inflammation and oxidative damage (Cai et al., 2022). Melatonin maintains mitochondrial homeostasis and integrity by activating the AMPK/PGC-1α/SIRT3/SOD2 pathway, effectively alleviating worsened renal IRI in obese rat models (Kobroob et al., 2023). The prostaglandin analog treprostinil improves the dynamic equilibrium of mitochondrial fusion and fission by restoring PGC-1α and SIRT3 expression (Ding et al., 2021). In drug repurposing strategies, alodipine enhances mitochondrial biogenesis by activating the NRF2/Sestrin2/PGC-1α/TFAM pathway (Shirzad et al., 2023). The aldosterone receptor antagonist eplerenone alleviates renal ischemia-reperfusion injury by maintaining mitochondrial function and promoting cell survival through upregulating the SIRT1/SIRT3/PGC-1α signaling pathway (Barati et al., 2023). Non-pharmacological interventions such as cold therapy and intermittent fasting also demonstrate the ability to inhibit fibrosis and cellular senescence processes by upregulating PGC-1α (Kim D. et al., 2025; Kim S. R. et al., 2025). Furthermore, the protective effect of the mtROS scavenger Mito-Tempo on TECs under oxidative stress conditions partially stems from its ability to restore TFAM protein levels (Zhao M. et al., 2021). Collectively, targeting PGC-1α and its associated pathways offers a novel therapeutic avenue for IRI-related renal injury.

##### 4.2.4.2 Targeting mitochondrial dynamics

The imbalance of mitochondrial dynamics in RIRI is mainly manifested as excessive fission and fusion block, which is the core link leading to apoptosis of renal TECs and renal dysfunction. Therefore, targeted regulation of the mitochondrial fission protein DRP1 and fusion protein OPA1 has become a promising therapeutic strategy. Currently, a variety of drugs and natural compounds have shown significant renoprotective effects in preclinical models by directly or indirectly modulating DRP1/OPA1 activity, expression, or post-transcriptional modifications.

The small molecule inhibitor Mdivi-1 can effectively reduce ROS generation, inhibit cytochrome C release and apoptosis by blocking the gtpase activity of DRP1, thereby improving renal function and tissue structure (Li et al., 2018). Peptide P110, which specifically blocks the interaction between DRP1 and Fis1, has been shown to significantly alleviate AKI in mouse and porcine RIRI models by reducing DRP1 mitochondrial recruitment and inhibiting the downstream cGAS-STING inflammatory pathway activation (Song et al., 2024). Emodin, a natural plant extract, can inhibit CamKII-mediated phosphorylation of DRP1, reduce fission level and attenuate apoptosis and necrosis after ischemia-reperfusion (Wang et al., 2022). Cordycepin could directly bind to DRP1 protein and inhibit its activity. In the renal fibrosis model, cordycepin reduces the secretion of IL-6 in renal TECs by inhibiting DRP1-mediated mitochondrial fission, thereby inhibiting the activation of fibroblasts, and ultimately reducing renal interstitial fibrosis (Sun et al., 2025). Secondly, endogenous molecules such as melatonin also show potential to regulate DRP1. Melatonin can inhibit fission and promote autophagy through the AMPK/DRP1 pathway, thereby reducing ROS levels and improving RIRI-induced inflammatory response and cell death (Wang et al., 2024). These results suggest that targeting DRP1-mediated fission has a clear protective role in RIRI prevention and treatment. However, it has also been suggested that DRP1-dependent mitophagy is, to some extent, necessary to remove damaged mitochondria and maintain mitochondrial quality control (Li et al., 2018). Therefore, how to achieve a balance between “inhibiting excessive fission” and “retaining moderate fission” is an urgent problem to be solved in future clinical translation.

In contrast to fission, mitochondrial fusion enhances mitochondrial function by maintaining the integrity of the membrane crest and network connectivity. OPA1 as a key protein for mitochondrial inner membrane fusion, is significantly downregulated in expression during RIRI, leading to mitochondrial fragmentation and energy metabolism disorders (Wang et al., 2019). Studies have shown that intervention measures that enhance the expression or activity of OPA1 have a significant renal protective effect. The SGLT2 inhibitor empagliflozin can promote mitochondrial fusion through the AMPK-OPA1 pathway in animal experiments and renal tubular epithelial cell models, reduce inflammatory responses, and achieve the effect of alleviating RIRI (Yang W. et al., 2023). Hyperoside can inhibit the activity of the stress protease OMA1, reduce its hydrolysis of OPA1, thereby maintaining more long-chain OPA1 (L-OPA1) forms, promoting mitochondrial fusion, and providing protection against IRI (Wu et al., 2019).

Some natural compounds synergistically regulate DRP1 and OPA1 to restore mitochondrial homeostasis through multiple mechanisms such as anti-oxidation and anti-inflammation. Curcumin can restore mitochondrial dynamics balance and inhibit cell apoptosis by activating NRF2/HO-1 antioxidant pathway, up-regulating OPA1 and down-regulating DRP1 expression in various AKI models (Cai et al., 2022). Schisandrin B can directly bind to AKT1 protein and promote its phosphorylation. The activated AKT1 signaling pathway can downregulate the expression of DRP1 and upregulate the expression of OPA1 and MFN2, thereby synergistically inhibiting fission and promoting fusion to improve mitochondrial function (Xu et al., 2025). Pharmacological therapeutic strategies targeting DRP1 and OPA1 have mainly focused on the preclinical stage of research, but their results have been fruitful, revealing the great potential of treating RIRI by restoring mitochondrial dynamic balance.

##### 4.2.4.3 Targeting mitophagy

Due to the core position of the PINK1/Parkin pathway in renal IRI, targeting this pathway has become a highly promising therapeutic strategy.

Platycodin D (PD), a compound extracted from the root of Platycodon grandiflorum, can activate AMPK and upregulate LC3B-II, PINK1 and Parkin, while inhibiting the MAPK/NF-κB inflammatory pathway, thereby alleviating diabetic renal IRI. Its protective effect can be completely blocked by the AMPK inhibitor Compound C (Qin et al., 2025). The muscle factor Irisin secreted during exercise can increase the expression of PINK1 and Parkin, reduce the levels of TOM20 and TIM23, and improve mitochondrial quality (Cui et al., 2024). In addition, the liver regeneration enhancer factor (ALR) also reduces ROS and NLRP3 inflammasome activation by activating PINK1/Parkin-dependent mitophagy, and its knockdown significantly aggravates mitochondrial dysfunction (Zhu et al., 2023). The regulation of non-coding RNAs also indicates the importance of PINK1/Parkin in the protection of IRI. Long non-coding RNA H19 was found in the *in vitro* ischemia-reperfusion model (CPB-AKI) to alleviate inflammatory responses and apoptosis by promoting PINK1/Parkin-dependent mitophagy, suggesting that it may become a potential molecular target (Zhang M. et al., 2025). MitoQ, a mitochondrial-targeted antioxidant, enhances mitophagy by activating NRF2 and restoring PINK1 expression (Xiao et al., 2017). Non-pharmacological interventions such as calorie restriction have also been proven to improve mitochondrial function and may activate autophagy/mitophagy, thereby enhancing renal ischemic tolerance (Andrianova et al., 2022).

Similar to the PINK1/Parkin pathway, BNIP3, as a member of the Bcl-2 family, also induces mitophagy in response to ischemia-related stress and participates in the pathological and protective processes of IRI. A growing number of studies have suggested that direct or indirect regulation of BNIP3 signaling can improve mitochondrial damage and cell death in IRI.

Oroxylin A (OA), an active ingredient extracted from Scutellaria baicalensis, was shown to maintain mitochondrial homeostasis, alleviate kidney injury and delay the progression of AKI to CKD in IRI and cisplatin AKI models by inducing PPARα-BNIP3 signaling pathway (Yao et al., 2022). 2-methoxyestradiol (2ME2), an endogenous metabolite of estrogen, was found to reduce the mRNA expression of TNF-α, IL-1β, caspase-9, HIF-1α and BNIP3, and increase the expression of anti-apoptotic proteins BCL-2 and BCL-xL, thereby reducing kidney injury (Chen et al., 2014). Yan et al. showed that exogenous spermine could exert a protective effect against renal IRI in rats by increasing the expression of HIF-1α and BNIP3 and inhibiting apoptosis of renal TECs (Yan et al., 2021). In the field of nanomedicine, cobalt oxide-polyethylene glycol triphenylphosphine (COPT) nanoparticles can dose-dependently activate BNIP3 mediated mitophagy, improve AKI and prevent its progression to CKD, providing a new idea for the intervention of BNIP3 pathway (Qin et al., 2023).

Overall, PINK1/Parkin and BNIP3 are the two most important mitophagy regulatory pathways in RIRI. The former mainly reduces apoptosis and inflammation by maintaining mitochondrial quality control and removing damaged mitochondria, while the latter mediates autophagy by regulating upstream signals such as HIF-1α and PPARα under ischemic environment, and shows duality under different experimental conditions. Pharmacological interventions, endogenous agents, and non-pharmacological measures have been shown to exert protective effects through these two pathways. In the future, further elucidation of the interaction between PINK1/Parkin and BNIP3 and exploration of its feasibility as a combined intervention target will provide new ideas for the transformation of IRI treatment strategies.

##### 4.2.4.4 Targeting mitochondrial metabolic reprogramming

A variety of therapeutic strategies targeting metabolic reprogramming have shown great therapeutic potential. These strategies mainly focus on restoring FAO, inhibiting abnormal glycolysis and improving mitochondrial function. In restoring fatty acid oxidation, application of PGC1α agonist ZLN-005 or AMPK activator could significantly enhance FAO and inhibit the transition from AKI to CKD, thereby preventing the transition from AKI to CKD (Xu et al., 2023). In diabetic IRI model, Dapagliflozin can effectively improve FAO dysfunction and inhibit abnormal glycolysis by activating SIRT3/PGC-1α signaling pathway, thus providing renal protection (Li H. et al., 2024). Similarly, the exercise hormone Irisin activates the AMPK/mTOR pathway by binding to the integrin αVβ5 receptor to correct the FAO/glycolysis imbalance and improve IRI (Zhao et al., 2025). In terms of inhibiting abnormal glycolysis, the natural compound Ovatodiolide inhibits the enzymatic activity and dimer formation of G6PD by specifically targeting its Lys403 site, thereby inhibiting the excessive activation of the pentose phosphate pathway and alleviating renal fibrosis (He et al., 2025). Inhibition of glycolysis with the hexokinase 2inhibitor 2-DG has also been shown to inhibit PMT and prevent the transition from AKI to CKD (Xu et al., 2023). In addition, emerging mitochondrial targeted therapies, such as the use of mitochondrial antioxidants (MitoQ, SS-31), mitophagy inducers (such as COPT nanoparticles) and even mitochondrial transplantation, have shown great promise in restoring mitochondrial bioenergetics, reducing oxidative stress and regulating inflammation (Pavlović et al., 2025). These studies show that metabolic reprogramming as a target to restore cellular energy metabolism homeostasis from multiple levels is a promising research direction for the prevention and treatment of RIRI and its development to chronicity. Metabolic reprogramming is not only the pathological product of RIRI, but also the driving force of its progression to CKD. Further understanding the metabolic remodeling patterns of different cells, such as renal tubules, endothelial cells and immune cells, through multi-omics methods and precision medicine strategies, may achieve effective clinical transformation.

##### 4.2.4.5 The clinical applicability and translational challenges

Although a variety of drugs and nanomaterials targeting mitochondrial dynamics and mitophagy have shown significant renal protective effects in animal experiments, their clinical application is still in its early stages, and there are still multiple challenges before they can truly enter the treatment of renal IRI. First of all, at present, most intervention drugs only remain in cell and rodent models, and the clinical evidence is extremely limited. For instance, the widely used Drp1 inhibitor MDIV-1 has issues such as insufficient target selectivity *in vivo* and potential impact on the functions of other GTPases. Its pharmacokinetics and long-term safety have not been verified, and thus it is not yet ready for human trials. Similarly, although natural compounds such as emodin, cordycepin, and curcumin have shown good effects in animal models, their low bioavailability, poor oral absorption, and limited stability *in vivo* all restrict their clinical transformation. In terms of nanomaterials, although nanoparticles such as COPT have demonstrated high targeting and significant efficacy in animal models, their clinical feasibility is still limited by the long-term toxicity of the materials, the methods of clearance *in vivo*, homing efficiency, and the need for standardized production processes. Nanoparticles may accumulate in the kidneys and induce unpredictable immune responses, all of which need to be verified through large animal experiments and strict toxicological evaluations. In addition, mitochondrial-targeted therapy faces two universal challenges: First, IRI is highly heterogeneous, with significant differences among various triggers (surgery, sepsis, transplantation) and metabolic backgrounds of different patients, making it difficult for a single intervention to be universally applicable. Second, both mitophagy and kinetics have a “double-edged sword effect” - moderate activation is protective, while excessive or insufficient activation may aggravate the damage. Therefore, clinical intervention requires precise control of the time window, dosage and regulatory degree, which poses extremely high demands on the design of the trial. Overall, therapeutic strategies targeting mitochondrial dynamics and mitophagy have a clear theoretical basis and strong clinical demand, but they still need to overcome key obstacles such as pharmacokinetic safety, targeting, individual heterogeneity, and bidirectional regulation. In the future, the combination of multi-omics analysis, patient stratification, large animal model validation, and the combined treatment strategy of drugs and nanomaterials will be an important direction for promoting its clinical transformation.

### 4.3 Key controversies and future challenges

Although mitochondria are recognized as central players in renal ischemia-reperfusion injury (RIRI), several key controversies and knowledge gaps persist, limiting both mechanistic understanding and clinical translation. A major controversy concerns the dual role of mitophagy. While timely removal of damaged mitochondria is protective, excessive or prolonged mitophagy under severe injury conditions can deplete mitochondria and worsen cellular energy collapse. This functional duality appears context-dependent, influenced by injury duration, cell type, and the precise “time window” of mitophagy activation. The regulation of mitochondrial dynamics also remains debated. Although inhibiting DRP1-mediated fission is often protective, fission itself is essential for mitochondrial quality control by facilitating the removal of damaged organelles. Therefore, simply suppressing fission may not always be beneficial. The net effect likely varies with pathological background—such as diabetes or aging—and injury stage, highlighting the need for more refined models to clarify these contextual dependencies. Furthermore, most current evidence derives from highly controlled rodent models, whereas clinical RIRI typically occurs in patients with comorbidities, polypharmacy, or altered immune status. Significant physiological differences between animal models and human patients limit the translational reliability of preclinical findings. Improving model standardization and developing more clinically relevant experimental systems are crucial steps toward future translation.

## 5 Limitations

Although this study combines bibliometric analysis and mechanism review to systematically present the development of mitochondria in the field of RIRI research from 2005 to 2024, there are still certain limitations. Firstly, the data sources mainly rely on the three major databases of Web of Science, Scopus and PubMed. Although they are widely covered and authoritative, gray literature, non-English literature and early research results that have not been digitized may still be omitted. The differences in inclusion strategies and update frequencies among various databases may also lead to certain time lags or coverage biases in research inclusion. Secondly, the bibliometric results are significantly influenced by the search strategy and parameter Settings, including keyword design, search formula construction, time window selection, as well as different Settings of thresholds and clustering algorithms in CiteSpace. These methodological choices may affect the results of network structure presentation, hot spot clustering, and emergent word analysis. Although we followed a relatively standardized process and presented the main parameters, there are still inevitable repetitive differences in the results. Finally, this study did not conduct a systematic assessment of the experimental quality of the included literature. RIRI animal models show significant differences in ischemia patterns, duration, animal strains, anesthesia protocols, and body temperature control. This high heterogeneity may affect the stability and comparability of mechanism research. However, bibliometric analysis is difficult to reflect these quality differences and may have a certain impact on the interpretation of hot trends. Although the above limitations may affect the comprehensiveness and extrapolation of the results, this study still provides an important reference for understanding the development trends of mitochondrial research in RIRI. Future research still needs to be further supplemented and verified by combining high-quality experimental data and clinical evidence.

## 6 Conclusion

This study is the first to systematically sort out and visualize the global field of mitochondrial research in RIRI through bibliometric methods. The results show that the field is in an active and rapidly developing stage. According to the results of keyword analysis, the mechanism exploration of mitochondrial dysfunction in RIRI, mitochondrial quality control and therapeutic intervention targeting mitochondria are the research hotspots. In addition, the classical pathways such as early apoptosis and oxidative stress have been gradually transformed into new concepts such as ferroptosis, mitochondrial quality control, and mitochondrial metabolic reprogramming. In conclusion, the central role of mitochondria in renal IRI has been established, and translating the mitochondrial protection targets identified in basic research into safe and effective clinical treatment options will be the greatest challenge and opportunity in the future. The knowledge map drawn in this study can provide valuable insights for relevant scholars to quickly grasp the overall situation of the field, locate research gaps, and choose cooperation directions.

## Data Availability

The original contributions presented in the study are included in the article/Supplementary Material, further inquiries can be directed to the corresponding authors.
