# Supplementary material for: Current perspectives and trends on the role of mitochondria in renal ischemia-reperfusion injury from 2005 to 2024: a bibliometric analysis and literature review
Source: Front Physiol. 2025 Dec 10;16:1705821. doi: 10.3389/fphys.2025.1705821 (PMC12727442; doi:10.3389/fphys.2025.1705821)
Supplement: Supplementary file 1 [file Table1.docx]

Literature retrieval strategy

Web of Science

#1: ((TS=(mitochondrial) OR TS=(mitochondria)) OR TS=(mitochondrion)

#2: (TS=(kidney)) OR TS=(renal)

#3: ((((TS=(ischemia* reperfus* injury)) OR TS=(lR injury)) OR TS=(IRI) OR TS=(ischemi* damage)) OR TS=(reperfus* injury)

#4: #1 AND #2 AND #3

Pubmed

(("ischemia reperfusion injury"[Title/Abstract] OR ("lR"[All Fields] AND "injury"[Title/Abstract]) OR "IRI"[Title/Abstract] OR "ischemia reperfusion injury"[Title/Abstract]) AND ("kidney"[Title/Abstract] OR "renal"[Title/Abstract]) AND ("mitochondrial"[Title/Abstract] OR "mitochondria"[Title/Abstract] OR "mitochondrion"[Title/Abstract])) AND (2005:2024[pdat])

Scopus

(TITLE-ABS-KEY(mitochondrial) OR TITLE-ABS-KEY(mitochondria) OR TITLE-ABS-KEY(mitochondrion)) AND (TITLE-ABS-KEY(kidney) OR TITLE-ABS-KEY(renal)) AND (TITLE-ABS-KEY(ischemia reperfusion injury) OR TITLE-ABS-KEY(ischemia-reperfusion injury) OR TITLE-ABS-KEY(lR injury) OR TITLE-ABS-KEY(IRI))AND PUBYEAR > 2004 AND PUBYEAR < 2025 AND ( LIMIT-TO ( DOCTYPE , "ar" ) OR LIMIT-TO ( DOCTYPE , "re" ) ) AND ( LIMIT-TO ( LANGUAGE , "English" ) ) AND ( EXCLUDE ( PUBSTAGE , "aip" ) )
